# High-level inducible Smad4-reexpression in the cervical cancer cell line C4-II is associated with a gene expression profile that predicts a preferential role of Smad4 in extracellular matrix composition

**DOI:** 10.1186/1471-2407-7-209

**Published:** 2007-11-12

**Authors:** Susanne Klein-Scory, Marc Zapatka, Christina Eilert-Micus, Sabine Hoppe, Elisabeth Schwarz, Wolff Schmiegel, Stephan A Hahn, Irmgard Schwarte-Waldhoff

**Affiliations:** 1 Department of Internal Medicine, IMBL, Knappschaftskrankenhaus, University of Bochum, Bochum, Germany; 2 Division of Viral Transformation Mechanisms, German Cancer Research Center, Heidelberg, Germany; 3 Department of Gastroenterology and Hepatology, Kliniken Bergmannsheil, University of Bochum, Bochum, Germany; 4 Department of Internal Medicine, Molecular Oncology, University of Bochum, Bochum, Germany; 5 Department of Theoretical Bioinformatics, German Cancer Research Center, Heidelberg, Germany

## Abstract

**Background:**

Smad4 is a tumour suppressor frequently inactivated in pancreatic and colorectal cancers. We have recently reported loss of Smad4 in every fourth carcinoma of the uterine cervix. Smad4 transmits signals from the TGF-β superfamily of cytokines and functions as a versatile transcriptional co-modulator. The prevailing view suggests that the tumour suppressor function of Smad4 primarily resides in its capability to mediate TGF-β growth inhibitory responses. However, accumulating evidence indicates, that the acquisition of TGF-β resistance and loss of Smad4 may be independent events in the carcinogenic process. Through inducible reexpression of Smad4 in cervical cancer cells we wished to shed more light on this issue and to identify target genes implicated in Smad4 dependent tumor suppression.

**Methods:**

Smad4-deficient human C4-II cervical carcinoma cells were used to establish inducible Smad4 reexpression using the commercial Tet-on™ system (Clontech). The impact of Smad4 reexpression on cell growth was analysed *in vitro *and *in vivo*. Transcriptional responses were assessed through profiling on cDNA macroarrays (Clontech) and validated through Northern blotting.

**Results:**

Clones were obtained that express Smad4 at widely varying levels from approximately physiological to 50-fold overexpression. Smad4-mediated tumour suppression *in vivo *was apparent at physiological expression levels as well as in Smad4 overexpressing clones. Smad4 reexpression in a dose-dependent manner was associated with transcriptional induction of the extracellular matrix-associated genes, BigH3, fibronectin and PAI-1, in response to TGF-β. Smad4-dependent regulation of these secreted Smad4 targets is not restricted to cervical carcinoma cells and was confirmed in pancreatic carcinoma cells reexpressing Smad4 after retroviral transduction and in a stable Smad4 knockdown model. On the other hand, the classical cell cycle-associated TGF-β target genes, c-myc, p21 and p15, remained unaltered.

**Conclusion:**

Our results show that Smad4-mediated tumour suppression in cervical cancer cells is not due to restoration of TGF-β growth inhibitory responses. Rather, tumour cell-ECM interactions may be more relevant for Smad4-mediated tumour suppression. C4-II cells with a high level inducible Smad4 expression may serve as a model to indicate further Smad4 targets responsive to diverse environmental stimuli operative *in vivo*.

## Background

Smad4 is a tumour suppressor gene primarily known for its frequent inactivation in gastrointestinal malignancies. Loss of Smad4 occurs in one half of pancreatic adenocarcinomas [1], in one third of metastatic colorectal cancers [2] and in every fourth carcinoma of the small intestine [3]. We have recently reported that Smad4 is also implicated in cervical carcinogenesis [4]. Four of 13 cervical cancer cell lines displayed Smad4 deficiency due to insertional inactivation or homozygous loss of 3' exons. Furthermore, Smad4 expression was lost in 10 of 41 primary squamous cervical carcinomas and reduced in 26 cases as shown by immunohistochemistry, whereas all cervical intraepithelial neoplasias (CIN) retained normal Smad4 expression levels.

Defining the mechanisms of Smad4 tumour suppressor function and identifying Smad4 target genes is critical to address its potential as a therapeutic or diagnostic target. Smad4 is characterised as a transmitter of signals of the TGF-β superfamily of cytokines. The founding member of this family, TGF-β1, potently inhibits the growth of normal epithelial cells. Cancer cells typically have lost sensitivity to TGF-β antiproliferative responses. When Smad4-deficient tumours were first identified, it was assumed that loss of TGF-β antiproliferative and proapoptotic responses underlie the tumour suppressor function of Smad4. However, during recent years it has become evident that the relationship of TGF-β and Smad4 is much more complex [5-11]. Accumulating evidence from our lab and others has suggested that Smad4 loss and TGF-β resistance attainable via multiple molecular alterations may be independent events.

Squamous cell carcinoma of the uterine cervix is typically initiated through infection with high-risk human papillomavirus types, in particular HPV16 and HPV18. The E6 and E7 viral proteins deregulate cell growth control through inactivation of the p53 and pRB tumour suppressor gene products. Again, the postulated association of Smad4 loss with TGF-β resistance appears less clear. Only few from the cervical carcinoma cell lines display some residual TGF-β responsiveness, irrespective of their Smad4-positive or Smad4-negative status [4]. Loss of Smad4 is a late step in gastrointestinal [12,2,13] as well as in cervical cancers [4], consistent with the hypothesis that Smad4 may function as a suppressor of invasion. TGF-β, in contrast, is characterised as a promoter of invasion in late stages of carcinogenesis [5-11]. Stable reexpression of Smad4 in colorectal and pancreatic carcinoma cells at physiological levels was adequate to suppress tumour growth *in vivo*, but did not restore TGF-β responsiveness [14,15]. Rather, we have shown that Smad4 regulates an angiogenic switch [15] and functions as a positive regulator of the invasion suppressor gene, E-cadherin [16,17], and of the heterotrimeric laminin-5 molecule, which is a major basement membrane constituent [18].

These data show that cell culture models are suited to investigate mechanisms underlying Smad4-mediated tumour suppression. However, it has been argued that stable transfection of Smad4 might select for clones that are resistant to the growth inhibitory function of Smad4 due to defects downstream of Smad4. Thus, we decided to generate stable transfectants from Smad4-deficient C4-II cervical cancer cells conditionally expressing Smad4. We have obtained clones with a range of expression levels for Smad4, spanning no expression, physiological expression and overexpression up to approximately 50-fold of the physiological level. Reduction of tumour growth in nude mice was observed in clones with either physiological or high Smad4 expression levels. In contrast, cell growth *in vitro *was not affected, except in clones with very high Smad4 overexpression. Resistance towards TGF-β-mediated growth inhibition, as measured by response of cell-cycle associated genes such as c-myc, p21 and p15, was retained in clones expressing physiological and 50-fold increased Smad4 levels.

We then used expression profiling to search for Smad4-dependent target genes. This revealed three genes, namely PAI-1, fibronectin and BigH3, which were induced in response to TGF-β in high as well as in low Smad4-expressing C4-II cells. Interestingly, all of these genes encode secreted proteins presumably affecting tumour matrix and stroma. It is notable that the basal expression levels (and TGF-β response) of these genes were also increased by Smad4 reexpression in Smad4-negative pancreatic carcinoma cell lines, and were reduced upon Smad4 knockdown in Smad4-positive cells.

## Methods

### Cell culture

C4-II cells were obtained from the American Type Culture Collection (Rockville, MD, USA). Cells were maintained in Dulbecco's modified Eagles Medium (DMEM) supplemented with antibiotics and 10% fetal calf serum (FCS) (Gibco). As Smad4 deficiency has not previously been reported for cervical carcinoma cells, we first checked the identity of the cell line. C4-II cells contain integrated HPV18 DNA. HPV18 specific transcripts were detected by RT-PCR. In addition, Southern hybridization of C4-II genomic DNA with an HPV18 specific probe yielded restriction fragments, which are indicative and specific for the HPV18 integration site in C4-II cells [4].

### Constructs

The set of vectors for tetracycline-inducible transgene expression were purchased from Clontech Laboratories. The full-length coding sequence of DPC4/Smad4 was derived from the pBK-DPC4 constitutive expression construct [14]. The DPC4/Smad4 coding sequence was inserted into the NheI/EcoRI sites of the pTRE-vector. The construct was confirmed by direct sequencing (Sequitherm Cycle Sequencing, Epicentre). All plasmids used for transfections were CsCl_2 _purified followed by proteinase K digestion, phenol extraction and precipitation.

Smad4 reconstituted pancreatic carcinoma cells were generated by retroviral transduction. The stable knockdown derivatives from human pancreatic carcinoma cell line, Paca44, were established through transfection of an siRNA construct kindly provided by F. Kanai [19,20].

### Conditional Smad4 expression in C4-II cells

C4-II cells were stably transfected using a standard calcium phosphate coprecipitation method [14] with the pTet-on™ plasmid encoding the reverse transactivator and the neomycin phosphotransferase gene. Transfectants were selected in media with 2 × 10^-4 ^g/ml G418 and single colonies were isolated and expanded. Clones that showed a low basal activity and high inducibility of the transactivator in the presence of doxycycline were identified by transient transfections with the TRE-luciferase reporter. Transient transfections were carried out with DAC 30™ transfection reagent (Eurogentec) according to the manufacturer's instructions. Luciferase was determined using a luciferase detection kit (Promega). Two clones displaying approximately 5-fold (clone 28) and 10-fold (clone 18) induced luciferase expression in transient assays were chosen for the second round of transfections. The TRE-DPC4 plasmid was cotransfected with pTK-Hyg or pY3-Hyg plasmids coding for the hygromycin B resistance gene. Selection was carried out in media with 2 × 10^-4 ^g/ml G418 and 15 × 10^-5 ^g/ml hygromycin B, yielding a total of 49 clones.

### Northern blot analysis

RNA was isolated by acid phenol extraction or using a commercial kit (RNeasy; Qiagen). Northern blots and hybridisations were performed as described [14]. Blots were stripped and reprobed for GAPDH as a loading control.

### Preparation of proteins and Western blot analysis

Cells were lysed in NP-40 lysis buffer (25 mM TrisHCl, pH 7.4, 0.5% NP-40, 100 mM NaCl, 1 mM EDTA) containing a protease inhibitor cocktail (Roche) and 1 mM PMSF. Protein lysates were subjected to standard SDS-PAGE and immunoblot analysis was performed as described [14]. The blots were incubated with monoclonal antibodies against Smad4 (anti-Smad4 B8; dilution 1:500, Santa Cruz). Antibodies against phospho-Smad2 and Smad3 from Biomol were used at a 1:1000 dilution.

### Transient transfection assays with p6SBE and p3TPlux reporter plasmids

The p3TP-Lux construct was a kind gift from J. Massagué [21]. The SBE-Luc reporter construct was kindly provided by B. Vogelstein [22]. C4-II cells were plated in 10 cm dishes. One day before transfection, half of the cells were treated with doxycycline (2 × 10^-6 ^g/ml) to induce Smad4 expression. Twenty-four hours later, 10^5 ^cells per well were plated in 24 well plates with or without doxycycline. Using the effectene transfection protocol, the cells were transfected with 2 × 10^-5 ^g p6SBE-luc reporter plasmid containing 6 Smad binding elements concatemerised in front of the reporter promoter or with 2 × 10^-5 ^g p3TPluc reporter plasmid containing AP1 and PAI-1 promoter elements. Transfection efficiency was determined from cotransfection of the β-gal reporter plasmid (4.5 × 10^-5 ^g per well). The transfection mixture was removed 24 h later, and the medium was replaced with fresh medium with or without TGF-β (5 × 10^-9 ^g/ml). The luciferase and galactosidase activities were measured after another 24 hours. All assays were done in triplicate.

### Analysis of TGF-β responses *in vitro*

For *in vitro *growth analysis 2 × 10^5 ^cells were plated on 60 mm dishes in medium with 0.5% fetal calf serum with or without 2 × 10^-6 ^g/ml doxycycline. Two days after plating medium was replaced with fresh medium with or without 5 × 10^-9 ^g/ml recombinant transforming growth factor beta 1 (R&D systems). Cell numbers were counted from duplicate plates every 3 or 4 days. Alternatively the cell cycle phase distribution was determined by flow cytometry after propidiumiodide incorporation.

For the analysis of endogenous transcriptional responses, 2 × 10^6 ^cells were plated on 60 mm dishes in standard medium or in serum-reduced medium (0.5% fetal calf serum) with or without doxycycline. TGF-β (5 × 10^-9 ^g/ml) was added after 24 hours, and cells were harvested for RNA preparations 1, 2, 4, 8 and 24 hours later. Where indicated, cells were treated with cycloheximide solubilised in 0.25% ethanol at a final concentration of 5 × 10^-6 ^g/ml.

### Tumour growth in nude mice

Suspensions of 5 × 10^6 ^cells in a volume of 0.1 ml of phosphate-buffered saline were injected subcutaneously into the flanks of 6-week old female athymic nude mice (Balb/c01aHsd-nu/nu). Smad4 expression was induced prior to injection of the cells through incubation in standard medium containing 2 × 10^-6 ^g/ml doxycycline, 1 × 10^-4 ^g/ml G418 and 75 × 10^-6 ^g/ml hygromycin B. To maintain Smad4 induction *in vivo*, drinking water for nude mice bearing doxycycline-induced cells was supplemented with 2 × 10^-3 ^g/ml doxycycline hydrochloride (Sigma) and 2.5% sucrose. Tumour growth was assessed every 3 days, and animals were sacrificed 2 weeks (clones 18-2 and derivatives) and 4 weeks (clones 28 and derivatives) after injection of the cells, when the largest tumour in the group reached a diameter of 10 mm. Tumours were excised, weighed and snap frozen. The tumour growth assays were performed twice with 3 mice per cell clone both with and without Smad4 expression. Statistical significance of tumour growth suppression was calculated using a t-test after Welch correction using Graph Pad Prism 4 software.

### Atlas hybridisation experiments

Macroarray hybridisation was performed on BD Clontech ATLAS human 1.2 microarrays according to the manufacturer's instructions. We used 3 × 10^-5 ^g total RNA from TGF-β- and doxycycline-treated cells for each hybridisation. The hybridised filters were analysed by phosphoimaging, and signals were quantified using GenePixPro software.

### Statistics

Data are presented as the mean and standard deviation. All experiments were done in triplicate and repeated at least twice with similar results. The numbers of mice per group are noted in the figure's legends. The two-tailed t-test with Welch correction was used to compare the tumor growth in nude mice. The p-values are indicated (significance p < 0.05).

## Results

### C4-II cells conditionally expressing Smad4 *in vitro *and *in vivo*

**Figure 1:**
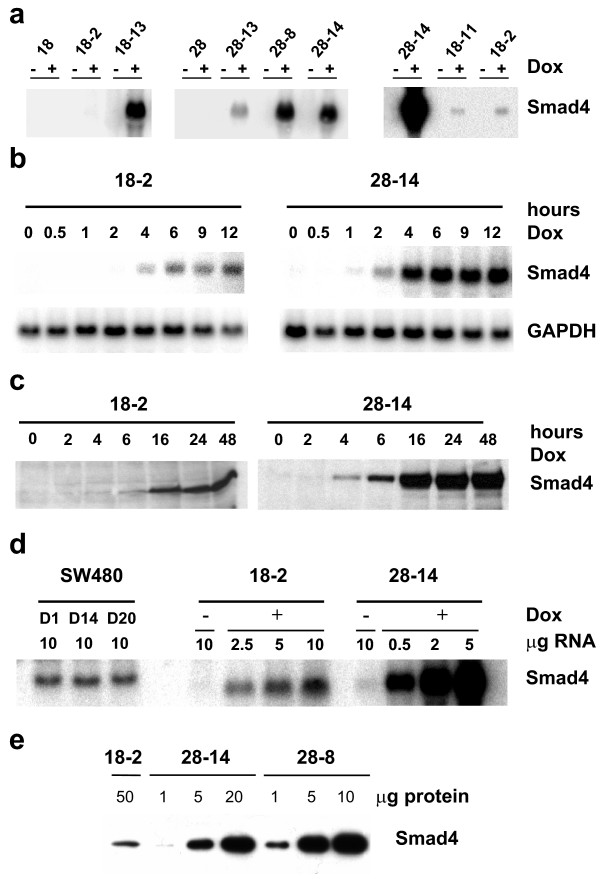
**Characterisation of inducible Smad4-reexpression in C4-II tet-on cell clones**. a) Northern blot showing Smad4-specific mRNA in a number of independent clones derived from tet-on clones 18 and 28. Exposure of the left and middle panel was for one day, exposure of the right panel was five days. b) Time course of Smad4-induction in the "low-expressor" clone 18-2 and in the high-expressor clone 28-14 at the mRNA level. c) Time course of Smad4-induction in clones 18-2 and 28-14 at the protein level. d) Titration of Smad4-mRNA levels in clones 18-2 and 28-14 as compared to Smad4-reexpressing SW480 cell clones. The latter clones have previously been shown to express (sub-)physiological Smad4 levels. e) Titration of Smad4 protein levels in clones 18-2, 28-14 and 28-8.

We chose to make use of the tetracycline conditional expression system using the original two-step protocol in order to avoid counterselection of Smad4-reexpressing clones due to putative growth inhibitory effects [23]. In a first transfection round, the tetracycline-dependent reverse transactivator was transferred and clones displaying inducible expression of a reporter gene under control of a tetracycline response element (TRE) were identified in transient transfections. Two „tet-on" clones, namely clone 18 and clone 28, were used in a second round of transfections with the TRE-Smad4 expression plasmid yielding a total of 49 derivative clones. We used Northern blot analysis to screen all clones, and identified 15 clones with inducible Smad4 expression and very low or absent background expression (examples are shown in Figure 1a).

The time-course of induced Smad4 expression was analysed in one clone, 18-2, with low Smad4 expression and in one clone, 28-14, with high Smad4 expression. Smad4-specific transcripts were detectable after 4 hours in clone 18-2, and as soon as 1–2 hours in clone 28-14. Maximal levels were reached after 6–9 hours (Figure 1b). As expected, Smad4 protein expression closely followed the time-course of mRNA induction (Figure 1c). The titration of RNA and protein revealed that the Smad4 expression level in clone 18-2 was approximately in the physiological range (similar to SW480 derivatives characterised in detail previously [14]) and that clones 28-14 and 28-8 expressed roughly 20- to 50-fold more Smad4 RNA and protein (Figure 1d, e). Thus, the inducible expression system allows investigation of Smad4 effects exerted at "normal" levels as well as upon strong overexpression.

### C4-II cell growth *in vitro *and *in vivo*

**Figure 2:**
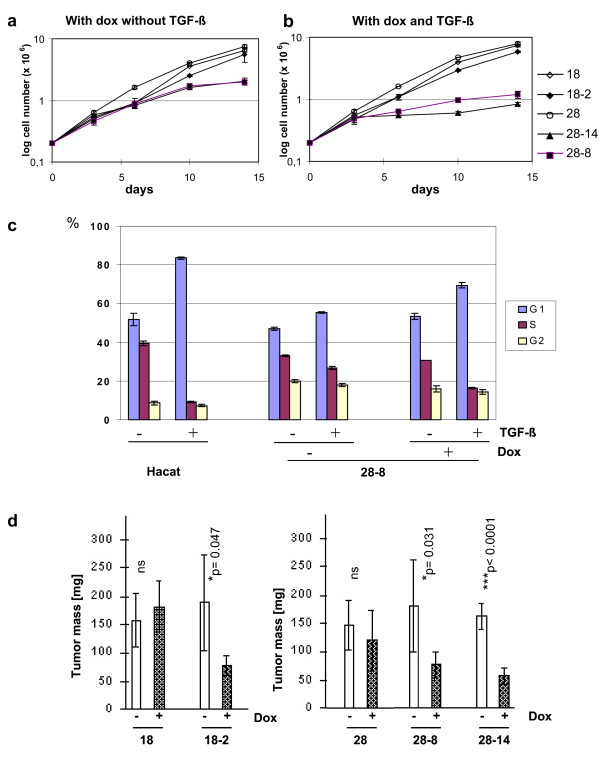
**Cell growth of Smad4-reexpressing C4-II tet-on cell clones *in vitro *and *in vivo***. Long-term growth curves of the tet-on clones 18 and 28 and Smad4-reexpressing derivatives 18-2, 28-8 and 28-14 were established in doxycycline-containing media in the absence (a) and in the presence (b) of TGF-β. c) Short-term TGF-β effects on cell cycle distribution were assessed in the high-expressor clone 28-8 as compared to HaCaT cells used as a positive control. Bars show mean values +/- standard deviation from three cultures per cell line and condition in one experiment. Similar results were obtained in repeated experiments. d) Cell growth *in vivo *was assessed by subcutaneous injection in nude mice. The open bars and hatched bars show the mean tumour mass +/- standard deviation of 6 tumours, each, after 2 (clones 18 und 18-2) or 4 (clones 28, 28-8 and 28-14) weeks of growth in mice that received drinking water without and with the addition of doxycycline. Statistical significance is indicated. Similar results were obtained in repeated experiments.

We have shown previously that Smad4-deficient C4-II cells do not respond to TGF-β [4]. This result was confirmed with the C4-II tet-on clones 18 and 28 (Figure 2a, b). Smad4 is known to mediate TGF-β-induced growth inhibition in normal epithelial cells. Thus, we wondered if inducible Smad4 expression was adequate to restore TGF-β responsiveness in these cells. Cell growth in standard medium was not affected by physiological Smad4 expression levels in clone 18-2, and addition of recombinant TGF-β did not exert a measurable effect (Figure 2a, b). In contrast, growth of clones 28-8 and 28-14 was moderately reduced in standard medium and upon long-term incubation, nearly came to a halt in TGF-β-containing medium (Figure 2b). Notably however, effects of TGF-β on the cell cycle distribution were moderate in 28-8 cells as compared to HaCaT cells, which served as a positive control [24] (Figure 2c).

Next, we analysed effects of Smad4 on cell growth *in vivo*. It has been shown previously, that expression of a transgene under the control of a TRE can be induced and maintained *in vivo *in mice by the addition of doxycycline to the drinking water [25]. To determine whether conditional Smad4 expression is functional *in vivo *in C4-II cell clones and is sufficient to mediate suppression of tumour growth, Smad4 expression was induced *in vitro *in one half of the cells by overnight incubation in doxycycline containing media. Cells were subcutaneously injected into the flanks of nude mice and doxycycline was added to the drinking water of mice bearing the induced cells. Tet-on clones 18 and 28 were included as controls and tumour growth was scored twice a week (Figure 2d). Addition of doxycycline to the drinking water did not affect tumour growth from tet-on clones 18 and 28, ruling out unspecific effects of the inducer. In contrast, mean tumour mass was significantly reduced to less than half as compared to the uninduced cells in the low expressor clone, 18-2, as well as in the high expressor clones, 28-8 and 28-14. Low and high Smad4 expression levels were retained in vivo, as confirmed in Northern blots of tumour-derived mRNA (data not shown). Thus, physiological Smad4 levels in this cell model are adequate to significantly reduce tumor growth.

### TGF-β responses in Smad4-deficient and in Smad4-reexpressing C4-II cells

The ability of Smad4 to mediate tumour suppression has largely been attributed to its presumed role as a mediator of TGF-β-induced growth inhibition. Our results do not support this hypothesis. Restoration of some TGF-β responsiveness was restricted to clones displaying 20- to 50-fold overexpression of Smad4, while physiological Smad4 levels were capable of exerting a Smad4-mediated reduction in tumour growth.

**Figure 3:**
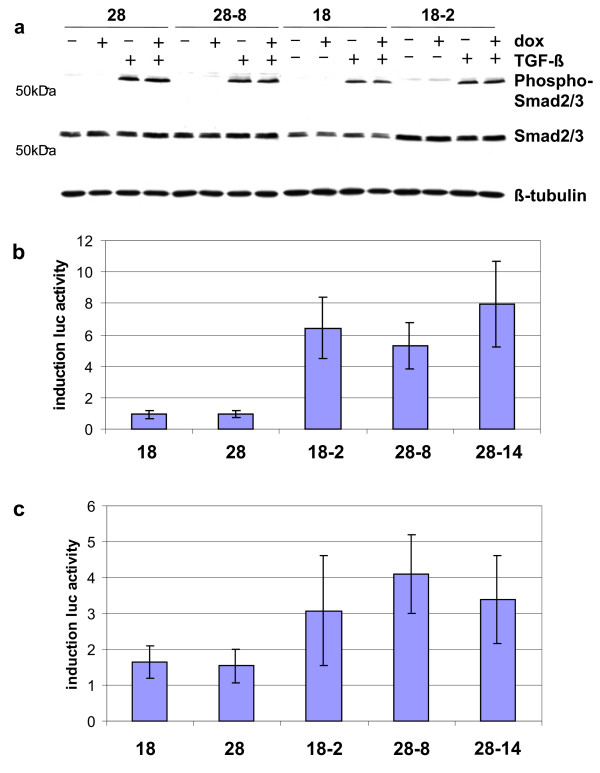
**Restoration of TGF-β responses in Smad4-reexpressing C4-II clones**. a) Phosphorylation of R-Smad2/3 in response to TGF-β was assessed through Western blotting with a phospho-Smad2/3-specific antibody. b) TGF-β response in transient transfections with the p6SBE-luc promoter; the induction factor in Smad4-reexpressing clones is 5–8. c) TGF-β response in transient transfections with the p3TP-luc promoter; induction in Smad4-negative cells is approximately 1.5 and rises to 3–4 in Smad4-reexpressing clones.

We, therefore, proceeded to characterise TGF-β responses in Smad4-deficient C4-II cells and in their Smad4-reexpressing derivatives in more detail. As the activation of TGF-β receptors might be limiting, we first analysed phosphorylation of receptor Smads, Smad2/3, in response to the addition of recombinant TGF-β (Figure 3a). No constitutive phosphorylation was observed, and phosphorylation in response to TGF-β did not differ between Smad4-deficient and Smad4-reexpressing clones. This result differs somewhat from published data, where high constitutive phosphorylation of receptor-Smads was reported in Smad4-deficient pancreatic cancer cells [26].

Next, we tested responses of the p6SBE and the p3TPluc promoter-reporter constructs frequently used to assess TGF-β responsiveness. The p6SBE promoter, constructed to reflect Smad-dependent transcriptional induction, contains concatemerised SBE (Smad binding element) sites [21,22]. As expected, luciferase activity of this promoter was not induced in the absence of Smad4 expression, but was strongly increased by a factor of 5–8 in Smad4-reexpressing clones (Figure 3b). The p3TPluc promoter is a hybrid promoter carrying sequences derived from the fibronectin and PAI-1 promoters optimised as an indicator of TGF-β responses. Interestingly, this construct was induced independently of Smad4 by TGF-β by a factor of approximately 1.5-fold in the tet-on clones (Figure 3c). As expected, all Smad4-reexpressing clones showed increased TGF-β induction of luciferase activity. Notably, induction levels in response to TGF-β using both constructs were quite similar between clone 18-2 and high-expressor clones, 28-8 and 28-14.

**Figure 4:**
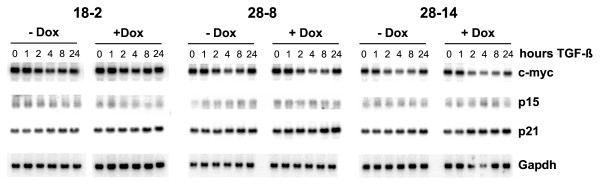
**Northern blot analysis of endogenous TGF-β target genes**. Clones 18-2, 28-8 and 28-14 were cultured in the absence or presence of doxycycline and treated with TGF-β for the indicated periods of time. Equal loading was controlled by hybridisation with a Gapdh probe.

As Smad4 proved fully functional in transmitting TGF-β responses in transient transfection assays, we then determined responses of endogenous TGF-β target genes (Figure 4). TGF-β-induced growth inhibition in normal epithelial cells is associated with Smad4-dependent alterations in cell cycle-associated target genes, namely the transcriptional repression of c-myc and induction of p15 and p21 [27,28]. Minor effects on the levels of c-myc transcripts were observed here, but were not dependent on Smad4 expression. No changes in the levels of p15 and p21 transcripts were detected (Figure 4). These results show that reexpression of Smad4, even at much higher levels, is not adequate to restore TGF-β-induced growth inhibition through the classical pathway.

### Smad4 effect on target genes encoding secreted proteins through indirect mechanisms

**Figure 5:**
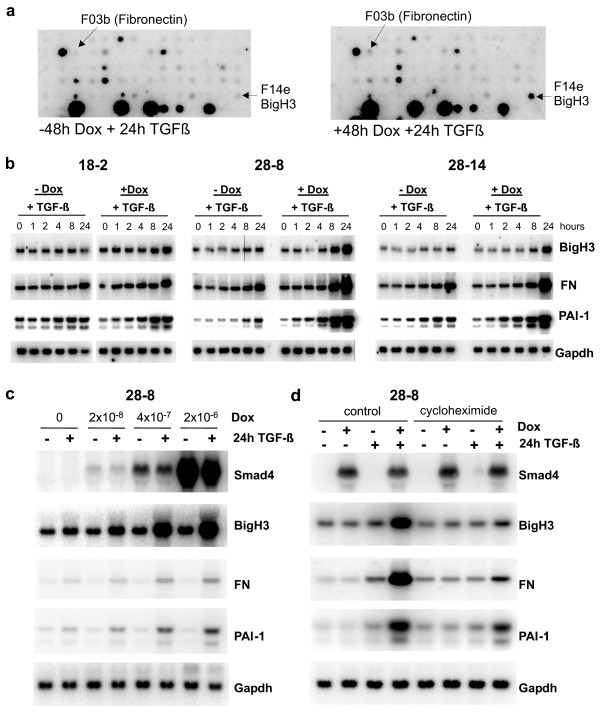
**Identification and validation of Smad4-dependent TGF-β target genes**. a) Smad4-dependent TGF-β target genes in 28-8 cells were identified through hybridisation of Clontech Cancer II arrays. At least three hybridisations were performed for each clone and each set of conditions. Shown as examples are sections of filters containing the probes for fibronectin and BigH3. The induction of PAI-1 was detectable but less clear because a very strong neighbouring signal faded over the PAI-1 locus (data not shown). b) Corresponding filters to those shown in figure 4 were hybridised with probes for BigH3, fibronectin and PAI-1. c) Smad4-levels were titrated in clone 28-8 through dilution of doxycycline. Northern blotting with the same probes as used in (b). d) TGF-β induction of 28-8 cells was repeated in the presence of cycloheximide

Smad4 target genes previously identified in colorectal and pancreatic carcinoma cells, namely E-cadherin [16], laminin-5 [18], VEGF and thrombospondin-1 [15], were not affected through Smad4 reexpression in C4-II cervical cancer cells (data not shown). We used expression profiling in order to detect alternative Smad4 target genes. Macroarrays were hybridised with cDNA derived from 18-2 cells and from 28-8 cells cultured in the absence or presence of doxycycline and untreated or treated with TGF-β. We detected no significant differences in expression profiles of 18-2 cells +/- doxycycline or +/- TGF-β. Likewise, 28-8 cells grown in standard media in the absence and presence of doxycycline did not display Smad4 target genes. Ultimately, hybridisation of cDNA derived from TGF-β-treated 28-8 cells revealed Smad4-dependent TGF-β induction of BigH3, fibronectin and PAI-1 (Figure 5a). It is noteworthy to mention that all of these target genes encode secreted and extracellular matrix-associated proteins. Northern blotting with RNA prepared from all three clones at different time points after TGF-β induction confirmed strong induction of these three target genes in the high-expressor clones, 28-8 and 28-14 (Figure 5b). The low expressor clone, 18-2, displayed significant but less-pronounced increases in mRNA levels of the target genes (Figure 5b). To examine if the effect was dose-dependent, we titrated Smad4 levels in clone 28-8. Also in this clone, low levels of Smad4 were sufficient to induce expression of BigH3, fibronectin and PAI-1 in response to TGF-β, and the extent of induction mounted in parallel with doxycycline and corresponding Smad4 levels (Figure 5c).

It has been shown previously, that TGF-β induction of fibronectin and PAI-1 can be mediated through a direct mechanism via activation of receptor-Smads and binding of heteromeric R-Smad/Smad4-complexes to SBE sites in the promoter regions of the genes. Here, induction of the three target genes was apparent only at late time points, suggesting that it might work through indirect mechanisms. In fact, cycloheximide treatment of the cells suppressed induction to virtually basal levels (Figure 5d).

These results show that TGF-β induction of BigH3, fibronectin and PAI-1 correlates with Smad4 expression levels and works via indirect mechanisms requiring protein synthesis.

### Smad4 control of secreted TGF-β target genes is not restricted to cervical cancer cells

In order to assess the scope of our findings we wished to know if Smad4 controls the expression of secreted TGF-β target genes in tumour cells derived from other organs. Smad4 was reintroduced into the BxPC3 and Capan1 pancreatic carcinoma cell lines via retroviral transduction, and led to approximately 3- to 5-fold Smad4 overexpression (data not shown).

**Figure 6:**
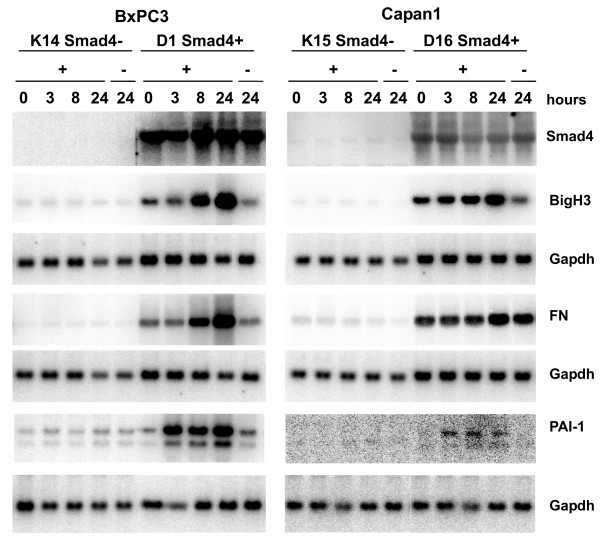
**Analysis of secreted TGF-β target genes in Smad4-reexpressing pancreatic carcinoma cells**. Stable reexpression of Smad4 was established in BxPC3 and Capan1 human pancreatic carcinoma cells through retroviral transduction. Shown are endogenous expression levels of BigH3, fibronectin and PAI-1 in response to TGF-β treatment of, a representative Smad4-deficient control clone and a Smad4-reexpressing clone from both cell lines.

Target gene analysis using Northern blotting revealed pronounced differences in basal expression levels of fibronectin and BigH3, and TGF-β, but all three target genes were induced in a Smad4-dependent manner (Figure 6).

**Figure 7:**
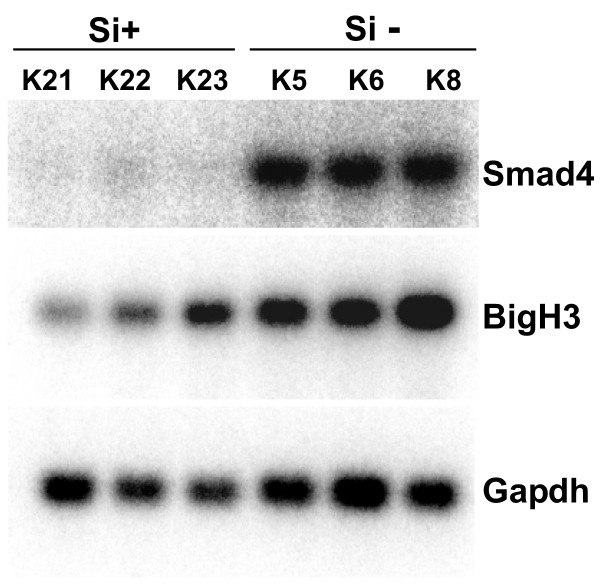
**Analysis of BigH3 expression**. Analysis of BigH3 expression in pancreatic carcinoma cells retaining endogenous Smad4 expression and upon stable Smad4-knockdown. RNAs from three independent Smad4-positive control clones and three knockdown clones, in which Smad4 expression was reduced to below 10% were hybridised with the BigH3 specific probe.

Lastly, we asked if Smad4 knockdown in Smad4 positive cells also would return the transcript levels of the secreted target genes to the basal level. To this aim we established stable derivatives from the human pancreatic carcinoma cell line, Paca44, via transfection of an siRNA construct kindly provided by F. Kanai [19,20]. Smad4 was downregulated to < 10% of the endogenous level in the resulting clones. Constitutive expression levels of BigH3 in Paca44 knockdown clones were significantly reduced (Figure 7). In addition, clones with reduced Smad4 expression exhibited reduced transcriptional responses to TGF-β (data not shown). These results provide additional evidence that Smad4 functions as a positive regulator of BigH3 expression in diverse cell types.

## Discussion

Concurrent with the identification of Smad4 as a tumour suppressor gene, the hypothesis was raised that loss of TGF-β antiproliferative and proapoptotic responses underlie the tumour suppressor function of Smad4. Stable Smad4 reexpression in colorectal and pancreatic cancer cells was adequate to mediate tumour suppression *in vivo*, did not inhibit cell growth *in vitro *and was not sufficient to rescue TGF-β antiproliferative responses. To rule out that stable Smad4 expression may select for clones in which TGF-β resistance is caused by additional defects downstream of Smad4, we developed a cell model with conditional Smad4 expression. We obtained clones using a tet-on system in which Smad4 can be reversibly turned off and on to levels ranging between zero and 50-fold of endogenous levels in the control cells. Using this system, we found that reduced tumour growth *in vivo *could be mediated through physiological as well as excessive levels of Smad4. In contrast, reduced cell growth *in vitro *and growth inhibition in response to TGF-β were restricted to cells strongly overexpressing Smad4. The "classical" cell cycle-associated TGF-β target genes were, however, not induced under these conditions, suggesting that growth responses in these cells *in vitro *do not reflect physiological mechanisms in cancer cells but occur only upon excessive Smad4 overexpression.

To the best of our knowledge, the only conditional Smad4 expression model published to date was developed by Calonge and Massagué [29]. These authors established cell clones derived from the SW480 colorectal carcinoma cell line using an ecdysone inducible system. They obtained maximal Smad4 expression levels which were comparable to endogenous levels expressed in HaCaT control cells. Consistent with our previous results using stable transfection of SW480 cells [14], conditional Smad4 expression also did not affect the TGF-β target genes, p21 and c-myc, and was not sufficient to restore TGF-β-induced growth inhibition.

We conclude from our results that, as has previously been shown for colorectal and pancreatic cancer cells [14,15], direct effects of Smad4 on tumour cell proliferation in cervical carcinoma cells are dispensable for Smad4-mediated tumour suppression. These results strengthen the idea that Smad4-dependent tumour suppression is due to more complex mechanisms functioning *in vivo*, which may include altered interactions of tumour cells with stromal cell types and the extracellular matrix. Target genes involved in the Smad4-mediated tumour suppression in colorectal and in pancreatic carcinoma cells, such as E-cadherin [16], laminin-5 [18], VEGF and thrombospondin-1 [15], however, did not respond to Smad4-reexpression in C4-II cervical cancer cells.

Expression profiling on macroarrays did not show altered expression levels of Smad4 target genes without TGF-β treatment of Smad4-expressing C4-II cells. After TGF-β treatment, the Smad4 target genes, fibronectin, PAI-1 and BigH3, were induced in C4-II cells expressing physiological levels of Smad4 and superinduced in Smad4-overexpressing cells. Whereas Smad4-dependent TGF-β induction of these genes is not novel [30-33], the underlying pathway appears to differ from that in normal epithelial cells. The linear TGF-β/Smad pathway signals from activated TGF-β receptors via phosphorylation of receptor-Smads. This leads to complex formation with Smad4, translocation of heteromeric Smad complexes into the nucleus and binding to SBE sites in the promoter regions of target genes. This direct pathway does not depend on protein synthesis, and results in rapid transcriptional induction. In Smad4-reexpressing C4-II cells, however, induction of the target genes appeared late and was suppressed to near basal expression levels by the addition of cycloheximide. We conclude that this Smad4-mediated induction requires protein synthesis, and may be executed through Smad4 induction of other transcription factors which in turn induce transcription of Smad4 target genes in an indirect manner. We used transient transfections of promoter-reporter constructs in order to identify putative transcription factor binding sites responsible for such indirect Smad4 effects. However, luciferase reporter activity in transiently transfected C4-II cells with or without doxycycline-induced Smad4 expression and with or without TGF-β did not reflect the transcriptional responses of the endogenous target genes. Unravelling the underlying molecular mechanisms in detail will require more cumbersome approaches. As Smad4-expression in C4-II cells can be tightly controlled, whole genome expression profiling in time-course experiments may indicate direct and indirect Smad4 target genes.

The three Smad4 target genes identified in C4-II cervical cancer cells encode secreted proteins which are deposited in extracellular matrices in tissues *in vivo*. It is well known, that interactions between cells and extracellular matrix (ECM) in general play a crucial role in tumour angiogenesis, invasiveness and metastasis [8,34]. Consequently, the roles of ECM molecules have been addressed in numerous studies. High expression levels of PAI-1, for example, have been correlated with pro- and anti-tumour effects. The reported prognostic relevance of PAI-1 in cervical cancer as well as in other cancer types is conflicting [35]. An expression analysis of BigH3 in cervical cancers has not yet been published. Interestingly, the expression levels of BigH3 in pancreatic carcinomas were reported to be high, although highly variable, in virtually every sample analysed in one study [36]. It would be interesting to correlate the Smad4 status of the tumour cells with TGF-β and BigH3 levels in this tumour panel.

Recent approaches addressing functions of BigH3 in the carcinogenic process have consistently suggested anti-tumour activity, and have unravelled novel mechanisms how this protein could exert tumour suppressive effects. BigH3, also known as keratoepithelin and as TGF-β-induced gene, is a ubiquitous constituent of the extracellular matrix [33]. It encodes a 69 kDa protein containing four internal fascilin I-like repeats (fas domains) and an RGD peptide at the C-terminus. BigH3 supports cell attachment and spreading, induces actin stress fiber formation, and binds fibronectin [37] and integrins. Skonier et al. have shown that BigH3 gene transfection into CHO cells led to a marked decrease in the ability of these cells to form tumours in nude mice [33]. Also, restoration of BigH3 in human bronchial epithelial cells resulted in a significant reduction in tumour growth [38,39]. BigH3 induced differentiation in keratinocytes [40] and inhibited proliferation and invasion in human neuroblastoma cells [41]. Interestingly, fastatin, the fourth FAS domain of BigH3, has been shown to induce apoptosis and suppress endothelial tube formation, thus inhibiting tumour growth through suppression of neovascularisation [42,43]. Although no reports concerning BigH3 functions in cervical tissues exist to our knowledge, it is reasonable to speculate that this protein may also exert anti-tumour effects in cervical tumours.

Smad4 functions as an integrator of cellular responses to multiple external stimuli. Consequently, the outcome of Smad4 reexpression on expression profiles of tumour cells *in vivo *will widely differ from expression patterns *in vitro*. The microenvironment *in vivo *is regulated through complex interactions between tumour and stromal cell types. Growth factors and cytokines other than TGF-β may be expressed either by the tumour cells, by activated fibroblasts, or by inflammatory and immune cells. Treatment with recombinant cytokines to mimic the effect of these stromal cell types *in vivo *in combination with whole genome expression profiling holds promise to discover additional Smad4 target genes in the future. C4-II cells conditionally expressing extensive Smad4 levels in such approaches may serve as superindicators of cytokine-induced Smad4-responsive genes.

## Conclusion

The heterologous reexpression of Smad4 in Smad4-deficient cancer cell lines could reduce tumor growth *in vivo*. The expression of classical TGF-β target genes involved in cell cycle regulation was not affected by Smad4 reexpression. Otherwise, Smad4 dose-dependently controls the TGF-β induced expression of genes associated with extracellular matrix. We conclude that Smad4 effects on the composition of extracellular matrix may underlie its tumor suppressive activity.

## Abbreviations

BigH3, TGFbeta induced gene H3; Dox, doxycycline; DPC4/Smad4, depleted in pancreatic carcinoma locus 4; FN fibronectin, GAPDH, Glyceraldehyde-3-Phosphate Dehydrogenase; HPV human papillomavirus; PAI-1, plasminogen activator inhibitor 1; TGF-β, transforming growth factor beta; TRE, tetracycline response element

## Competing interests

The author(s) declare that they have no competing interests.

## Authors' contributions

SK-S participated in the design of the study, established the inducible expression system, carried out mouse experiments and contributed to writing the manuscript, MZ performed the atlas expression analysis and established Smad4-reexpressing pancreatic cancer cells, CE-M characterised the inducible C4-II cell clones, performed macroarray hybridisations and Northern and Western blot analyses, SH performed the analysis of pancreatic cancer cells. ES participated in the molecular characterisation of C4-II cells and edited the manuscript. WS contributed to the design of the study. SAH generated the inducible smad4 expression plasmid and retroviral expression vectors, IS-W is the PI, designed the study and drafted the manuscript.

## Pre-publication history

The pre-publication history for this paper can be accessed here:


